# Beta Cell Therapies for Preventing Type 1 Diabetes: From Bench to Bedside

**DOI:** 10.3390/biom10121681

**Published:** 2020-12-16

**Authors:** Gabriel Brawerman, Peter J. Thompson

**Affiliations:** 1 Department of Physiology and Pathophysiology, Rady Faculty of Health Sciences, University of Manitoba, Winnipeg, MB R3E 3P4, Canada; umbraweg@myumanitoba.ca; 2 Children’s Hospital Research Institute of Manitoba, Winnipeg, MB R3E 3P4, Canada

**Keywords:** pancreatic beta cells, type 1 diabetes, apoptosis, senescence, immunotherapy

## Abstract

Type 1 diabetes (T1D) is a chronic metabolic disease characterized by insulin deficiency, generally resulting from progressive autoimmune-mediated destruction of pancreatic beta cells. While the phenomenon of beta cell autoimmunity continues to be an active area of investigation, recent evidence suggests that beta cell stress responses are also important contributors to disease onset. Here we review the pathways driving different kinds of beta cell dysfunction and their respective therapeutic targets in the prevention of T1D. We discuss opportunities and important open questions around the effectiveness of beta cell therapies and challenges for clinical utility. We further evaluate ways in which beta cell drug therapy could be combined with immunotherapy for preventing T1D in light of our growing appreciation of disease heterogeneity and patient endotypes. Ultimately, the emergence of pharmacologic beta cell therapies for T1D have armed us with new tools and closing the knowledge gaps in T1D etiology will be essential for maximizing the potential of these approaches.

## 1. Introduction

### 1.1. Autoimmune Type 1 Diabetes

Autoimmune type 1 diabetes (T1D) mellitus (also referred to as type 1A diabetes) results from insulin deficiency due to autoimmune-mediated destruction of pancreatic beta cells [1]. It often is distinguished from the less common type 1B diabetes, or idiopathic/nonautoimmune diabetes, in which the latter shows insulin deficiency and beta cell loss in the absence of beta cell autoimmunity [2]. T1D incidence has been increasing worldwide for the past few decades [3,4], and while it has a long-standing reputation as a pediatric disease, more recently an increasing number of young adults have been diagnosed [1,3]. Children and youth with T1D often experience challenges with insulin dosing to maintain optimal glycemic control as they age. Hence long-term diabetes-related complications such as nephropathy, neuropathy and retinopathy may be observed earlier in their lifetimes [1]. Additionally, despite modern improvements in insulins, which have increased the healthy lifespan of people living with T1D, there are growing financial barriers to affordable access in many countries [5,6] and an overall high risk of cardiovascular disease development, which is a major cause of death among older people living with T1D [7]. Currently there is no way to prevent or cure T1D, and daily insulin administration is the only safe and effective way to manage the disease. Thus, in addition to ongoing clinical care for those living with the disease, there is also an urgent need to better understand T1D pathogenesis and develop preventive therapies and treatments. 

### 1.2. Stages in T1D Pathogenesis

Clinical heterogeneity in T1D patients is thought to arise as a result of different environmental exposures during development and genetic factors, each of which play a significant role in precipitating beta cell autoimmunity [3,8]. Models for the natural history of T1D have been extensively refined over the past decades as a consensus view has emerged [9,10,11]. In brief, three distinct clinical stages of disease progression have been recognized, although the timing and onset of each varies. During the earliest disease stage, patients are asymptomatic and as a result of environmental trigger(s) and genetic susceptibility, they develop beta cell autoimmunity (seroconversion) indicated by the presence of one or more autoantibodies against beta cell antigens, commonly insulin (INS), glutamate decarboxylase 65 (GAD65), islet antigen 2 (IA-2) and zinc transporter 8 (ZNT8) [12]. Remarkably, this early asymptomatic stage can precede a T1D diagnosis for years, and an increased number of autoantibodies correlates well with increased risk for T1D onset [13,14]. Newly developed risk scores incorporating genetic, epidemiological and immunological factors are greatly increasing predictive power for T1D onset in children between the ages of two to eight years old [15]. Stage 2 is characterized by declining beta cell function and/or mass as evidenced by abnormal glucose tolerance and sometimes very mild hyperglycemia [1]. However, overt hyperglycemia and the classical diabetes symptoms of polydipsia, polyuria and polyphagia are absent. Recent evidence suggests that beta cell dysfunction, rather than exclusively loss of beta cell mass during this period, may be a critical factor for disease progression [16]. Eventually, in the third stage, patients progress to become fully symptomatic, where functional beta cell mass is insufficient to meet normal metabolic demands leading to persistent hyperglycemia and the classical diabetes symptoms with or without diabetic ketoacidosis.

Interestingly, a honeymoon period has been described in an estimated 50% of new onset pediatric patients where their symptoms seem to improve and they experience clinical remission of diabetes upon the first administration of insulin and the subsequent reduction in dosage [17]. However, this phase is inevitably short-lived, usually lasting a few months, and patients subsequently require insulin again. This phenomenon is very poorly understood, but may suggest avenues for the proper timing of therapies to recover beta cell function in the long term after diagnosis [18]. Remarkably, although it was once believed that all beta cells are destroyed in T1D, recent work indicates that even in established T1D cases (>three years after diagnosis), proinsulin secretion persists for years in nearly all patients [19], and a substantial proportion of beta cells remain in many patients [20,21,22,23]. These observations hold promise for efforts to restore beta cell function well after diagnosis.

### 1.3. T1D as a Disease of the Immune System and Beta Cells

Historically, T1D has been viewed a disease of the immune system [24] where the beta cells are passive targets destroyed by a complex autoimmune process mediated by self-reactive cytotoxic CD4^+^ and CD8^+^ T cells supported by innate immunity. As a result of this emphasis, clinical interventions for the prevention and treatment of T1D have focused on immune-targeting therapies, some of which have shown beneficial effects [25,26]. For example, a recent clinical trial using a nondepleting anti-CD3 antibody (teplizumab), which targets T cells, in relatives of patients with T1D who were themselves at high risk of developing the disease (≥ 2 auto-antibodies and early signs of dysglycemia) led to a ~three year median delay in the progression to T1D onset when compared to a placebo [26]. However, the exact mechanisms of action of teplizumab are still unclear, and this therapeutic antibody also had little effect in some patients (e.g., non-responders) [26]. Similarly, a recent trial using golimumab, a therapeutic monoclonal tumor necrosis factor alpha (TNF-α) antibody, led to increased residual beta cell function and reduced insulin usage in new onset pediatric and young adult patients with T1D as compared with placebo [27]. This study also reported an increased number of hypoglycemic events along with increased frequency of infections in the golimumab patients [27]. Thus, while some immunotherapies can delay disease progression during stage two or even after onset in stage three, there are patients who do not respond and there are often unintended consequences of systemic immune modulation. A wide variety of immunotherapy clinical trials for new onset T1D or T1D prevention/delay are underway and include immune-modulating antibodies, cytokines, vaccines and regulatory T cell therapies [28].

Building from the classical paradigm of T1D as an autoimmune disease, a growing body of evidence supports the idea that beta cell dysfunction is just as critical as the autoimmune process, and that T1D is also a disease of the beta cells/islets [28,29]. Genome-wide association studies (GWAS) indicate that the majority of polymorphisms outside of the human leukocyte antigen (HLA) complex that are associated with T1D reside in genes known to be expressed in beta cells, including the *INS* gene itself [30]. Clinical observations over the past several years support the notion of ongoing beta cell dysfunction prior to diagnosis, and persistent beta cell mass and function, even in established T1D years after diagnosis [19,20,21,31,32,33]. Thus, an new emphasis on beta cell drug therapies could be an exciting avenue to reduce beta cell death, restore beta cell function and avert T1D onset during stage two or early into stage three of the disease [16]. In this review, we focus on the some of the mechanisms that mediate distinct forms of beta cell dysfunction during stage two and stage three at T1D onset as supported by evidence from both mouse and human studies, including beta cell apoptosis, senescence and other dysfunctional states and highlight clinical translation efforts and opportunities for targeting these pathways. We also discuss the potential to combine beta cell therapies with immunotherapy for T1D prevention in light of the ongoing re-evaluation of T1D etiology, which will be essential for maximizing the effectiveness of each type of therapy.

## 2. Beta Cell Dysfunction in T1D 

### 2.1. Beta Cell Endoplasmic Reticulum Stress, Unfolded Protein Response and Apoptosis

**Figure 1:**
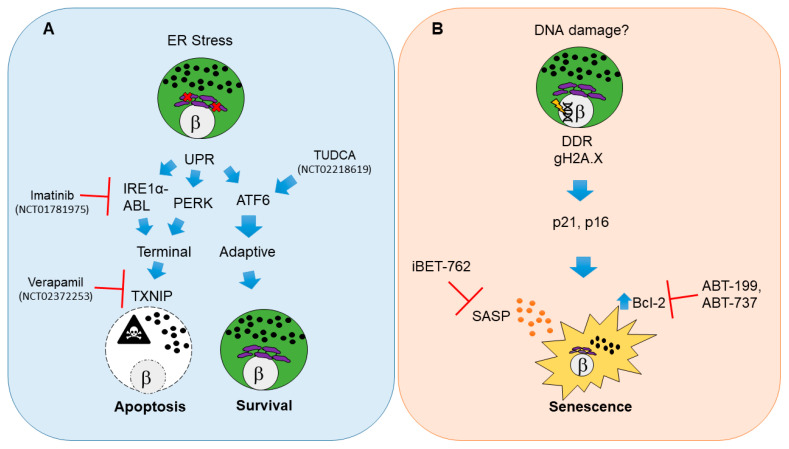
Molecular pathways and therapeutic targets for beta cell unfolded protein response (UPR)-mediated apoptosis and senescence in type 1 diabetes (T1D). (**A**) Beta cell apoptosis in T1D results from persistent endoplasmic reticulum (ER) stress that leads to activation of UPR master regulators IRE1α, PERK and ATF6. IRE1α mediates its functions through its RNAse and kinase activities that are potentiated by the Abelson tyrosine-protein kinase (ABLs). The balance of each UPR regulator dictates the outcome on beta cell fate. Unrelieved ER stress signals through IRE1α and PERK and shifts the pathway towards a terminal UPR and apoptosis mediated by thioredoxin interacting protein (TXNIP), whereas ATF6 is the major mediator of adaptive UPR leading to beta cell survival. Clinical trials in new onset adult T1D patients have used Verapamil, Imatinib or tauroursodeoxycholic acid (TUDCA) to attenuate terminal UPR and apoptosis and/or enhance adaptive UPR to delay the decline in residual beta cell function. (**B**) Beta cell senescence in T1D may be initiated by unresolved DNA damage (although the precise triggers of DNA damage remain unknown). A persistent DNA damage response (DDR) in beta cells is indicated by gH2A.X which is mediated by ATM. DNA damaged beta cells show activation of cyclin-dependent kinase inhibitors p21 and p16, which enforce a senescent growth arrest. Senescent beta cells upregulate the antiapoptotic protein Bcl-2 and develop a senescence-associated secretory phenotype (SASP). Small molecule inhibitors including senolytic compounds targeting Bcl-2 (ABT-199, ABT-737) or suppressing SASP at the level of gene expression (iBET-762) mitigate the deleterious effects of accumulated senescent beta cells in NOD mice and prevent T1D. These drugs have not been tested in clinical trials for T1D. The white circles and the β symbol indicate the nucleus, while the purple structure is the ER and black dots indicate insulin granules.

Perhaps the most well studied and widely regarded state of beta cell dysfunction during the pathogenesis of T1D is endoplasmic reticulum (ER) stress leading to apoptosis [34] (Figure 1A). Apoptosis is a form of programmed cell death triggered via a variety of mechanisms including internally as a result of irreparable cellular damage (intrinsic pathway), or externally as a result of surface receptor interactions with immune cells (extrinsic pathway) or as a result of the perforin-granzyme pathway [35] (for a detailed review of cell death mechanisms and nomenclature see [36]). 

As beta cells have high demands for insulin synthesis, processing, folding and secretion, metabolic and immune-mediated stress are believed to directly impact the ability to sustain these processes [23]. As a consequence, a major cause of apoptosis in beta cells is ER stress-mediated activation of the unfolded protein response (UPR) [37]. Accordingly, decreased *Ins1* gene dosage transiently improves beta cell ER function and relieves basal UPR stress in mice [38]. The UPR is a three-branched system that can either enable cells to maintain homeostasis (adaptive UPR) or lead them to commit to apoptosis (terminal UPR) [39]. Adaptive UPR signaling allows beta cells to cope with the stress of unfolded/misfolded proteins in the ER and recover, whereas a terminal UPR occurs if the stress is too great or prolonged, triggering apoptosis [40] (Figure 1A). Although recent work has focused on terminal UPR signaling as the major mechanism driving beta cell apoptosis, evidence from the widely studied nonobese diabetic (NOD) mouse model for human T1D [41] suggests that beta cells also undergo apoptosis via a combination of both the extrinsic pathway and perforin-granzyme pathway directed by cytotoxic T cells [42,43,44,45]. Similarly, a number of studies on human donor pancreas tissue have supported the idea that beta cells are destroyed in a heterogeneous fashion over the pancreas by CD8^+^ T cell-mediated cytotoxicity [20,21,33,46,47]. 

Another form of beta cell death suggested to be involved in T1D is necrosis [34], a lesser known form of cell death resulting from extensive damage, where cells are lysed and cellular contents are extruded to the extracellular environment, provoking immune activation and inflammatory responses [48]. This contrasts with what is thought to occur during apoptosis, as apoptotic cells are typically very short-lived and eliminated by phagocytes, leading to tissue remodeling [35,48]. While ongoing necrotic beta cell death is an attractive explanation for autoantigen release as has been proposed [49,50], the evidence for necrotic beta cell death as a putative mechanism in T1D is inconclusive. 

A major open question in this area concerns which forms of beta cell death predominate in T1D, and whether beta cell death is generally continuous, relapsing-remitting or purely situational and context dependent [51]. Interestingly, a recent study using DNA methylation as a biomarker for circulating cell-free DNA (cfDNA) originating from beta cells found no evidence to substantiate ongoing beta cell death (where death was measured as beta cell-derived cfDNA in serum) in seroconverted individuals or those with recent onset or established T1D, whereas the same bioassay was sensitive enough to detect beta cell death following islet transplantation [52]. Thus, the various forms of beta cell death during the development of T1D, whatever they may be, either differ from those during islet transplantation or are simply not continuously occurring. As our knowledge of cell death mechanisms continues to expand [36], it will be important to elucidate additional pathways of beta cell death in T1D.

#### 2.1.1. Pathways for UPR-Mediated Beta Cell Apoptosis

In beta cells, adaptive and terminal UPR are held in balance downstream of ER stress. ER stress activates the tripartite UPR pathway involving master regulators inositol requiring enzyme 1 alpha (IRE1α), PKR-like ER Kinase (PERK) and activating transcription factor 6 (ATF6), each of which regulate processes that control the apoptotic versus survival fate decision [37]. Notably, mRNA and protein markers of ER stress and UPR activation in beta cells are apparent in early-stage prediabetic NOD mice and human T1D donor pancreas sections [53,54,55]. When ER stress is prolonged or beyond remediation, there is a shift from adaptive to terminal UPR via IRE1α or PERK-dependent activation of the redox protein thioredoxin interacting protein (TXNIP) in beta cells [40,56] (Figure 1A). TXNIP activation is necessary to trigger the intrinsic apoptotic pathway in beta cells [40,56]. Accordingly, terminal UPR and apoptosis in beta cells can be averted with small molecule inhibitors targeting the RNAse activity of IRE1α or its binding partner, Abelson tyrosine-protein kinase (ABL) [57,58]. Recent genetic evidence indicates that IRE1α also controls beta cell identity, and beta cell-specific knockout of this UPR mediator protects against T1D in NOD mice [59]. Similarly, *Txnip* knockout prevents induction of apoptosis in rodent beta cell lines and islets ex vivo under conditions of persistent ER stress, such as high glucose [60]. 

#### 2.1.2. Clinical Trials for UPR Therapies in T1D

Clinical trials testing beta cell-directed drug therapies for T1D (e.g., where beta cells are the primary target of the experimental agent, excluding transplantation) are few and far between. As of November 2020, on the clinicaltrials.gov website there were over 2100 listed interventional T1D trials (inclusive of all trial statuses), yet only around 100 of these involve beta cells as drug targets, most of which are repurposing drugs currently used to treat type 2 diabetes (T2D). Interventional clinical trials utilizing small molecule drugs (alongside standard insulin regimens) to mitigate beta cell apoptosis in T1D in adults (≥ 18 years old) have shown promising early results in small cohorts. A recent phase II placebo-controlled clinical trial using daily Verapamil (TXNIP inhibitor) in recent onset T1D in adult patients over 12 months (NCT02372253) demonstrated enhanced preservation of beta cell function, reduced hypoglycemic events and decreased insulin requirements [61] (Figure 1A). However, the study size was quite limited (n = 11 participants for each treatment group) and also reported a high frequency of gastrointestinal side-effects and nausea, making it unclear whether this drug would be tolerable in a pediatric population. Similarly, a recent clinical trial using daily imantinib (IRE1α-ABL inhibitor) in recent onset T1D patients (NCT01781975) demonstrated a partial preservation of beta cell function compared with placebo over a one-year follow-up period (study has not been published) (Figure 1A). However, a broad range of adverse side-effects were reported and occurred more frequently in the imatinib-dosed group, categorized as gastrointestinal, skin, respiratory, cardiac, endocrine and infectious, suggesting a wide range of off-target effects. Indeed, it was recently reported that in addition to terminal UPR signaling, imatinib also directly affects insulin secretion from beta cells [62] and promotes reactive oxygen species (ROS) scavenging by B cells in NOD mice, an effect which is essential for diabetes reversal [63]. Thus, it would seem that there is still much to learn about the mechanisms of this drug.

Terminal UPR and apoptosis may also be averted by enhancing the ability of beta cells to handle unfolded proteins. The bile acid derivative tauroursodeoxycholic acid (TUDCA) acts as an ER stress inhibitor and protein chaperone [64] and prevents diabetes in the NOD mouse model in an ATF6-dependent manner [54] (Figure 1A). Notably, TUDCA-related acids have been safely used in infants and children for some time now as a treatment for various hepatobilliary diseases [65,66], suggesting they would also be safe for pediatric patients. A placebo-controlled phase II clinical trial for TUDCA in recent-onset adult T1D patients (NCT02218619) has recently completed, although the results await publication. The findings of this study will be important in providing further clinical evidence for the potential of UPR inhibitor therapies to enhance beta cell survival and function in T1D. 

While these studies are indeed promising, a key feature of each of these drug therapies is that they require continuous dosing to inhibit their targets and be effective (daily dosing regimens were used in each of these trials). This kind of regimen often maximizes the extent and severity of side-effects. Indeed, uptake of these UPR inhibitory drugs in cell types other than ER-stressed beta cells would be detrimental when terminal UPR and apoptosis are required for tissue regeneration and cell turnover. Nevertheless, the evidence from these clinical trials suggests that even at diagnosis there is a clear window to improve, or at least delay, the decline of residual beta cell function beyond insulin therapy alone. The question of whether beta cell function can be improved in T1D by repurposing T2D drugs remains open. However recent studies targeting glucagon-like peptide 1 (GLP-1) and GLP-1 receptor (GLP1R) signaling suggest that this may not be effective (NCT01155284, NCT02284009) [67,68]. As future studies begin to understand the points at which beta cells are most vulnerable to ER stress-induced functional decline and terminal UPR during the various stages of T1D development, it may be possible to use these therapies intermittently and when they are most needed, obviating the side-effects resulting from chronic daily administration.

### 2.2. Damage-Induced Beta Cell Senescence

Non-lethal forms of beta cell dysfunction also contribute to T1D development. A subpopulation of beta cells in the late-stage prediabetic NOD mouse, in seroconverted asymptomatic donors and recent onset and established human T1D donors activate a DNA damage-induced senescent fate [69] (Figure 1B). Senescence is a form of programmed growth arrest, often triggered by various types of irreparable cellular damage, aging or oncogene activation [70]. While senescence is classically viewed as a single state or phenotype, a growing body of literature supports the notion that there are different kinds of senescence depending on cell type, developmental stage and triggers [71,72,73,74,75], and the kinds of senescent cells that accumulate in various tissues during aging have pathogenic effects of tissue physiology [76,77,78]. On the other hand, beneficial forms of senescence are employed for a variety of essential processes, such embryonic patterning [72,79], tissue regeneration [75], wound healing [80] and tumor suppression [81]. Thus senescence has been suggested as an example of antagonistic pleiotropy during evolution [82,83]. The lack of a single universal marker of senescence in vivo has made the accurate phenotypic definition of these cells in various tissues very challenging. Thus multiple independent markers are typically necessary to substantiate claims of senescence [84].

The triggers of the initial DNA damage and senescence induction in beta cells during T1D remain to be determined. Nevertheless, the observation that beta cell senescence and apoptosis both occur during the pathogenesis of T1D in humans and mice is consistent with the fact that they are both damage-induced fates [85]. What is it that leads some beta cells to commit to the terminal UPR, while others activate a damage-related senescence program? Beta cells are known to be heterogeneous at the transcriptional and functional levels [86,87,88,89,90,91] and heterogeneity occurs on multiple levels in T1D [3]. Addressing this fundamental question about heterogeneity in beta cell fates will be of great importance for understanding T1D pathogenesis.

#### 2.2.1. Molecular Pathways of Damage-Induced Beta Cell Senescence 

In the context of T1D, damage-induced senescent beta cells show hallmarks of a persistent DNA damage response (DDR) involving Ser139 phosphorylated histone H2A.X (also called gamma-H2A.X) [69], which is typically elicited by the master kinase ataxia telangiectasia mutated (ATM) and marks double-strand breaks [92]. The senescent growth arrest is effected in these cells by the upregulation of classic cyclin-dependent kinase inhibitors, cyclin-dependent kinase inhibitor 1a (Cdkn1a, also called p21) and Cdkn2a (encoding both p19^Arf^ and p16^Ink4a^) [69]. ATM activation usually signals to induce *Cdkn1a* expression via the tumor suppressor protein p53 and knockout of *Atm* in beta cells attenuates the DDR that is activated by the DNA-damaging agent streptozotocin [93] confirming the conservation of this pathway in beta cells. Notably, the form of beta cell senescence in T1D is distinct from what is observed during age-related senescence in beta cells [94,95] and the senescence in T2D [96]. Aged beta cells upregulate p16^Ink4a^ but not p21 and do not show evidence of ongoing DNA damage [69,94,95]. The persistent DDR of senescent beta cells in T1D also distinguishes them from the form of beta cell senescence in T2D, which resembles an accelerated aging phenotype [23,96]. Furthermore, it should be noted that senescence is not exclusive to beta cells in T2D and the related metabolic syndrome, but occurs in multiple cell types including preadipocytes and hepatocytes [97,98,99]. A similar senescence signature to NOD mice was also observed in human beta cells in a small cohort of seroconverted donors (single or double autoantibody positive), recent onset and established T1D donors (spanning < 1 year to six years disease duration) [69]. Senescence in human beta cells in T1D is apparently related to DNA damage, as supported by the observation that similar senescence markers can be induced in normal human islets in culture with the DNA damaging agent bleomycin [69]. Notably, an earlier report also found evidence for activation of the DDR in beta cells of new onset T1D donors (weeks to a few months after diagnosis), indicated in that study by foci of the repair factor p53 binding protein 1 (53BP1) [93]. 

Damage-induced senescent beta cells develop two additional phenotypes, particularly relevant to their deleterious effects on the islet microenvironment and T1D progression. First, they selectively upregulate the antiapoptotic protein B cell lymphoma 2 (Bcl-2) [69] (Figure 1B). Bcl-2 family members are pro or antiapoptotic, dictating a finely-tuned control mechanism over intrinsic apoptosis [100]. Upregulation of the antiapoptotic family members, including B cell lymphoma extra-large (Bcl-xL), B cell lymphoma w (Bcl-w) and/or Bcl-2 seems to be a major hallmark of most forms of senescence and confers a prosurvival phenotype in senescence and cancer [100,101]. Thus, senescent beta cells can potentially evade the external cues from their environment, including from infiltrating lymphocytes and resident inflammatory macrophages that would otherwise trigger apoptosis. This feature in particular sets damage-induced beta cell senescence apart as a totally distinct fate compared with UPR-activated apoptosis, as senescent beta cells are long-lived. Second, senescent beta cells can activate a proinflammatory secretome typical of other kinds of senescent cells, and originally referred to as the senescence-associated secretory phenotype (SASP) [69,102,103]. SASP is a context-dependent and dynamic program of secreted cytokines, chemokines, growth factors, shed receptors and matrix proteases that are highly immunogenic and mediate paracrine signaling with neighboring cells [70,74,104]. The main purpose of SASP in vivo seems is to be immune surveillance and clearance of senescent cells from the tissue, leading to resolution of inflammatory responses [84,104]. However, in the context of T1D, SASP seems to go unresolved as senescent beta cells continue to accumulate during disease progression [69]. Senescent beta cells also have elevated lysosomal β-galactosidase activity [69], a phenotype common to beta cell aging and beta cell senescence in T2D [95,96], referred to as senescence-associated β-gal activity [105].

It remains to be determined what causes the transition from senescence to SASP in beta cells, as only a subset of the senescent beta cells apparently develop SASP markers, and there is great variation in the frequency of SASP beta cells in NOD mice and humans donors with T1D [69]. Finally, it is important to note that while these accumulated senescent beta cells show alterations in some key beta cell identity genes (such as decreased Ucn3) [69], they are distinct from beta cells that become fully dedifferentiated (e.g., showing elevated endocrine precursor marker Ngn3) or transdifferentiated (e.g., showing a bihormonal or polyhormonal phenotype). This conclusion is supported by the observations that they maintain high *Ins1* and *Ins2* expression based on single-cell RNA-seq and have apparently normal levels of insulin content by immunohistochemistry [69]. Whether the senescent beta cell subpopulation in NOD mice overlaps with the subset that resists autoimmune attack and persists during established diabetes in this model [106], remains to be determined, although the putative antiapoptotic phenotype of the former is consistent with this idea.

#### 2.2.2. Potential for Clinical Translation of Senescence-Targeting Therapies in T1D 

Pharmacologically, senescent beta cell accumulation can be mitigated, leading to a halt in the autoimmune process and prevention of T1D in NOD mice. Bcl-2 inhibitors that act as senolytic compounds selectively induce apoptosis in senescent beta cells (Figure 1B) without any detectable alteration in the major lymphoid or myeloid cell types in T1D [69]. Thus, treatment of isolated islets from NOD mice or administration of senolytic compounds ABT-199 or ABT-737 to prediabetic mice diminishes the expression of senescence and SASP markers ex vivo and in vivo [69]. Notably, ABT-199 (also called Venetoclax) was recently approved by the Food and Drug Administration as a first-in-class Bcl-2 inhibitor for combination treatments in chronic lymphoid leukemias where Bcl-2 is overexpressed. Similarly, pharmacologic suppression of SASP in beta cells is achieved by transcriptional inhibition of the bromodomain extraterminal domain (BET) protein family [107]. Small molecule BET inhibitor iBET-762, currently in phase I/II trials for various cancers [108], prevents diabetes and suppresses SASP in beta cells of NOD mice in vivo and in human islets ex vivo [107]. An earlier generation BET inhibitor iBET-151 was also shown to prevent T1D in NOD mice, and indicated effects on both beta cells and macrophages [109]. Taken together, these findings imply that while BET inhibitors suppress SASP in beta cells, they also dampen BET protein-mediated inflammatory pathways in myeloid cells [110]. Nevertheless, evidence from studies in NOD mice, human pancreas donor specimens and islet culture models supports the clinical potential of beta cell senescence therapies for T1D prevention. It remains unclear whether senescence-targeted therapy would be beneficial after T1D onset or could be useful during the partial T1D remission honeymoon phase. 

In order to successfully move senescence-targeted therapies towards a clinical context, there are certain challenges that need to be overcome. First, the current generation of senescence-targeting drugs and senolytics are repurposed from the oncology field, and while most have acceptable side-effects profiles in adults, they have not been tested in children and thus might pose significant risks. Open-label small cohort phase I trials to therapeutically eliminate senescent cells in adult patients with diabetic kidney disease [111] or idiopathic pulmonary fibrosis [112] have used a senolytic cocktail of dasatinib and quercetin (D+Q) administered intermittently, and have demonstrated good safety and some efficacy. But it is unclear whether D+Q would affect the senescent beta cells that accumulate in T1D. Second, as these drugs all have off-target effects, it will be necessary to develop targeted delivery approaches to ensure maximal uptake by senescent beta cells. Emerging approaches for therapeutic targeting of senescent cells in other tissue sites [113] might be informative for developing such a system for beta cells. Finally, the lack of clinical correlates hinders the ability to predict which seroconverted patients have the highest burdens of senescent beta cells and thus would stand to benefit most from this therapy. Indeed, there seems to be wide variation in the frequency of senescent beta cells in islets of recent onset T1D and seroconverted donors [69], underscoring the notion of heterogeneity in beta cell fates. A biomarker for senescent beta cells would set the stage for interrogating patient cohorts to establish relationships between senescence and other clinical parameters to identify patients that would be good candidates for beta cell senescence therapy [69].

### 2.3. Other States of Beta Cell Dysfunction: Defective Proinsulin Processing and Bihormonal Beta/Islet Cells

Substantial evidence for other nondestructive dysfunctional states in beta cells has been recently reported. These include defects in proinsulin processing in established T1D [19,114,115], and transdifferentiation/altered identity in recent onset and established T1D [116,117]. Proinsulin is the precursor molecule following removal of the N-terminal signal peptide from preproinsulin in the ER [118]. Neuroendocrine peptidases prohormone convertase (PC) 1 and 3, PC2 and carboxypeptidase E (CPE) catalyze sequential proteolytic cleavage events that ultimately generates mature insulin and C-peptide for exocytosis [119]. Notably, independent studies have now demonstrated a proinsulin processing defect in established T1D, indicated by (1) increased proinsulin-to-insulin ratio in islets, and (2) persistent proinsulin secretion detected in the serum of long-term T1D patients [19,114,115]. *PCSK1* mRNA (encoding both PC1 and 3 isoforms) was significantly decreased in T1D pancreata, whereas expression of *PCSK2* (encoding PC2) and *CPE* were not affected [115], suggesting that the defect in proinsulin processing arises as a result of diminished PC1/3 activity. Another study confirmed this finding at the protein level, with reduced PC1/3 detected from T1D donor islets and a trend towards decreased CPE levels [114]. Additionally, while *INS* mRNA was abundant in established T1D pancreata, very little nascent transcript (referred to as heterogeneous nuclear RNA) was detected from the *INS* promoter, suggesting that ongoing *INS* transcription is disrupted in T1D [115]. How, when and why the proinsulin processing defects arise in long-standing T1D are important questions for future studies. This will permit therapeutic approaches to improve proinsulin processing and possibly insulin production and secretion in long-standing T1D patients. Whether the state of defective proinsulin processing is a feature co-occurring with UPR and/or senescence in beta cells, remains to be determined.

In addition to proinsulin processing defects, a subset of beta cells in recent-onset and long-standing T1D have also been shown to adopt a bihormonal state, with concurrent production of the alpha cell hormone glucagon in addition to insulin [116,117]. The idea that islet cells transdifferentiate in T1D was initially unsubstantiated when Lam et al. (2017) stained pancreas specimens from a large cohort of T1D donors spanning children to older adults with various disease durations (from new onset to established) for islet endocrine markers and found no evidence of new beta cell formation (termed neogenesis) or bihormonal islet cells [22]. However, another study published around the same time identified a very minor subpopulation (2–5%) of islet cells in a small cohort of established T1D donors that were double-positive for glucagon and insulin, but lacked canonical alpha cell identity markers Aristaless related homeobox (ARX) and DNA methyltransferase 1 (DNMT1) [117]. Subsequently, an improved histochemical staining approach was developed to identify very low-level insulin expressing cells (Insulin^Low^) in islets from recent-onset and established T1D donors, which were suggested to represent the histological correlate to the clinical persistence of insulin micro-secretion in long-standing T1D [32,116]. Prior work had suggested that a subset of beta cells become insulin-negative but retain pan-endocrine marker chromogranin A [120,121], consistent with a loss of beta cell identity. However, the more recent study [116] suggested that improved staining methods could reveal whether some of the previously defined insulin-negative islet cells may actually be Insulin^Low^. 

Notably, Insulin^Low^ islet cells were found in T1D donors of every age, indicating this phenotype is not correlated to disease duration, and a subset of these cells in recent and established disease were shown to co-express beta and alpha cell transcription factors homeobox protein NKX6.1 and ARX, respectively [116]. Whether these are beta cells that had transdifferentiated, or alpha cells that had acquired low level insulin production and beta cell identity markers, could not be determined. However, other islet endocrine cell hormones were also reported in the Insulin^Low^ cells, including somatostatin, ghrelin and pancreatic polypeptide, indicating that Insulin^Low^ cells are not arising exclusively from alpha-to-beta interconversion [116]. Do these cells arise during the asymptomatic stages and play a causal role in T1D pathogenesis, or are they a later consequence of the metabolic effects of and sub-optimal glycemic control? Additional studies are clearly necessary to delineate the origins of Insulin^Low^ cells in T1D pancreata and determine whether insulin production and beta cell identity can be restored to these cells in T1D patients.

### 2.4. Additional Mechanisms of Beta Cell Dysfunction

There are several other mechanisms that may contribute to various forms of beta cell dysfunction, which remain poorly understood, including antiviral responses and defects in autophagy and mitochondrial function. While definitive evidence of a viral etiology for T1D remains to be formally established [8], many studies have associated viral infections with T1D [122]. Indeed, many T1D GWAS loci reside in genes with known antiviral functions, mediating innate immune signaling via the type I interferon pathway [123]. Antibody-mediated suppression of type I interferon signaling prevents T1D in NOD mice [124], and emerging therapies targeting type I inteferon signaling are being deployed to combat a variety of systemic autoimmune diseases [125]. HLA class I hyperexpression occurs during the pathogenesis of T1D [126] and has been linked to type I interferon signaling in human islets and EndoC-βH1 beta cell models [127]. Polymorphisms in genes encoding innate immune and antiviral factors walk a fine balance between an effective host response to viral pathogens on the one hand, and the precipitation of autoimmunity on the other [128]. Interestingly, interferon signaling also promotes expression of programmed death ligand 1 (PD-L1) on beta cells in NOD mice and humans [129,130] a key immunoprotective factor on beta cells [131], thus future therapeutic interventions to promote beta cell survival might exploit this pathway. 

Beta cell autophagy is another important mechanism necessary for ensuring survival during conditions of stress in mice and humans [132,133,134]. A recent preprint study identified defective autophagy in beta cells of T1D pancreas donors relative to healthy controls [135]. Other beta cell organelles that may undergo functional impairment include mitochondria [136]. While a recent study suggested no major ultrastructural alterations in the mitochondria of beta cells in a small cohort of T1D donors by electron microscopy (EM) [137], a new large repository of EM imaging data from a much larger sample of nondiabetic, autoantibody-positive and T1D donors [138] will be useful for elucidating the frequency of structural defects in beta cell mitochondria during the development of T1D. 

## 3. Combining Beta Cell Therapy with Immunotherapy for T1D Prevention

### 3.1. Strengths and Weaknesses of a Combination Therapy Approach to T1D Prevention

**Figure 2:**
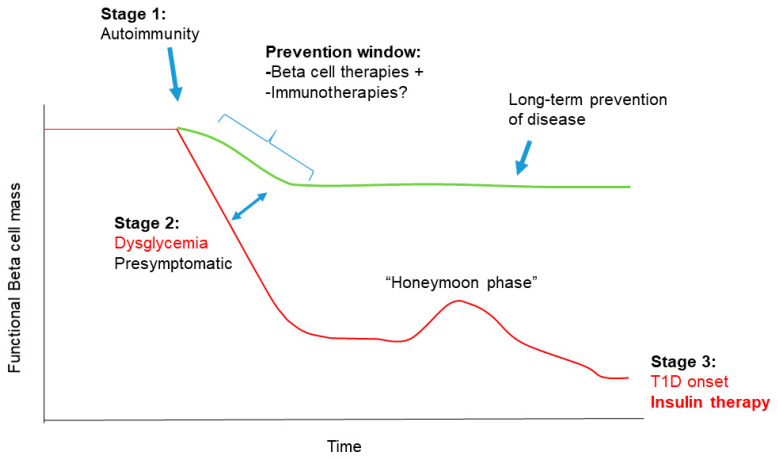
Combining beta cell therapy and immunotherapy for T1D prevention. There is a clear window for preventing T1D onset during stage 2, where seroconversion and dysglycemia are evident but patients are otherwise asymptomatic. The effectiveness of beta cell-targeted therapies, such as drugs inhibiting UPR or targeting senescence could be synergistic with immunotherapy during this stage. Intermittent use and more targeted delivery of these treatments during this preventive window could afford long-term prophylaxis against further loss of beta cell mass and function (green line), altering the typical trajectory of declining beta cell mass and function leading to T1D onset (red line).

The idea of combining existing beta cell therapies with immunotherapies for treating T1D has been recently advocated [28], as it presents an attractive approach for effectively addressing dysfunction on both sides of the disease pathogenesis [139] (Figure 2). The concept could involve therapies targeting terminal UPR in combination with immunotherapies, such as CD3 antibodies, with the intention of both improving beta cell survival and dampening or reversing beta cell autoimmunity during the window after seroconversion and initial onset of metabolic dysfunction (stage two). As we continue to discover the subtle immune and metabolic changes that accompany the progression of serconversion, this window in the natural history affords an opportunity for interventions that could prevent further decline of functional beta cell mass (Figure 2). However, the use of combination therapies would not be without its challenges in the clinic. Each of the therapies alone present a distinct set of risks for adverse side-effects. Therefore, combining therapies would greatly increase the frequency of these events in a given patient cohort. It is possible that intermittent treatment regimens and more targeted drug delivery could mitigate adverse side-effects to some extent. Moreover, beta cell therapy and immunotherapy could be administered in an alternating manner, as there is no reason to suggest that administering both forms of treatment together would be required for optimal efficacy. Nevertheless, a further limitation to implementing combination therapy in T1D could stem from the unpredictability of the effects of the one therapy on the other cell type (e.g., effects of beta cell therapy on the immune system). For example, ER stress and UPR inhibitor imatinib, which seems to partially delay functional beta cell decline in new onset T1D (NCT01781975) and spares beta cells to reverse T1D in NOD mice [57] also acts on ROS signaling in B cells, a feature which is necessary for its therapeutic effects [63] as mentioned above. Predicting and disentangling the unintended effects of beta cell therapies on cells in the immune system could, therefore, present a major obstacle for moving forward with evidence-based clinical trials using combination approaches.

### 3.2. Combination Therapy and the Re-Evaluation of T1D Etiology

Perhaps even more relevant to the potential utility of combined therapy is the presupposition that views T1D as a single uniform entity (albeit involving both immune and beta cell components). This perception is clearly losing traction, as there is an increasing appreciation for interpatient variability in virtually every aspect of the disease from epidemiology and potential environmental triggers to age of onset, sex differences, aggressiveness of autoimmunity, metabolic derangements and insulin efficacy [3,47]. Our current knowledge gaps in T1D etiology, and the relative contributions of beta cell death and dysfunction on the one hand, and immune system dysfunction on the other, are areas where investigators are becoming more critical of long-held assumptions [29]. Indeed, some in the field are now calling for a total re-evaluation of T1D etiologies based on the concept of disease endotypes, involving predominantly immune versus predominantly beta cell-driven pathogenesis [139,140,141]. Accordingly, the field has already recognized other distinct, but as yet poorly understood forms of insulin-deficient T1D. These span a spectrum of highly aggressive autoimmunity in fulminant T1D [142] to the absence of beta cell autoimmunity in idiopathic/nonautoimmune T1D [2]. Somewhere between these extremes, and possessing features of both T1D and T2D, is latent autoimmune diabetes in adults (LADA) [143,144], which manifests much later in life than classical T1D. LADA shows evidence of beta cell dysfunction and/or loss in the presence of mild autoimmune development, necessitating changes to typical T1D care regimes [145].

Although still largely conjectural at this point, accumulating experimental and clinical evidence in support of an endotype framework in autoimmune T1D will pave the way for personalized interventions and improving the effectiveness of clinical trial design [141]. With such knowledge, it would then be possible to select the most suitable therapies based on patient endotype, a major step towards a personalized medicine approach long dreamt of for this disease [141,146]. However, even if patients could be accurately stratified according to T1D endotype early in the natural history (e.g., during stage two, Figure 2), the future would entail a more specific approach of clinical trials using single agent therapy (e.g., beta cell or immunotherapy) tailored for the distinct endotype rather than attempting to affect both beta cells and the immune system with combination therapy. At any rate, as clinical trials for beta cell-targeted drug therapies in T1D are still in their infancy, as compared with the staggering number and history of immunotherapy trials [28,147], it is unlikely that beta cell therapy will be combined with immunotherapies for preventing T1D in the near future.

## 4. Conclusions and Future Directions

The emerging shift in paradigm of viewing T1D as purely an autoimmune disease to a heterogeneous disease of both the immune system and islets, is a profound one that has already furnished new therapeutic opportunities. Beta cell UPR, senescence, proinsulin processing defects and identity changes are all avenues with strong potential for developing long term preventive approaches for those at risk of T1D onset. Indeed, these states may even be just the tip of the iceberg, as there is no reason to suppose that there are no other forms of beta cell dysfunction yet to be discovered in T1D. There is also a growing recognition of dysfunction in other islet cells such as alpha cell glucagon secretion [148] and exocrine atrophy and pathophysiology [149,150], which may also provide therapeutic targets.

Clearly many questions remain to be addressed in this area. What is the relationship between these beta cell dysfunctional states, and what is it that causes beta cells to adopt one versus another in a given islet and patient? Which ones co-occur or are mutually exclusive? What are the fundamental causes of each, and what bearing do they have on disease pathogenesis at the clinically defined stages? Will it be possible to combine beta cell therapies with one another to target different forms of beta cell dysfunction simultaneously? These are all critical questions for the field to address in the future if our knowledge of beta/islet cell dysfunction in T1D can be safely and effectively translated to the clinic. It is clear that our understanding of T1D will continue to evolve and be refined due to advancements in experimental tools and approaches, such as high sensitivity immunohistochemistry [116], single cell phenotyping [87], image cytometry and high-throughput analysis [20,21], ultrasensitive hormone assays [19] and pancreas slice technology [47]. But progress will also depend on the willingness to challenge dogma and long-held assumptions about T1D [24,140,141]. It is on this foundation that the promise of therapies aiming to restore beta cell function and survival for preventing and treating T1D will eventually be fulfilled.
